# Rearing characteristics of fattening Hanwoo steers managed in different stocking densities

**DOI:** 10.5713/ajas.17.0451

**Published:** 2017-08-22

**Authors:** Jae Jung Ha, Ka Young Yang, Dong Yep Oh, Jun Koo Yi, Jong Joo Kim

**Affiliations:** 1 Gyeongbuk Livestock Research Institute, Yeongju 36052, Korea; 2 Animal Environment Division National Institute of Animal Science, Wanju 55365, Korea; 3 Department of Biotechnology, Yeungnam University, Gyeongsan 38541, Korea

**Keywords:** Growth Performance, Behavior, Carcass Trait, Stocking Density, Hanwoo

## Abstract

**Objective:**

This study was conducted to analyze the effects of stocking density on growth and carcass quality, and behavior of Hanwoo cattle to conform with global trends, targeting animal welfare production through the practice of environmentally friendly condition.

**Methods:**

Thirty six steers were randomly assigned to three treatment groups (C: 5 heads, T1: 4 heads, T2: 3 heads) and reared in separate pens with a constant stocking density of 50 m^2^ (C: 10 m^2^/head, T1: 12.5 m^2^/head, T2: 16.67 m^2^/head) per group from 12 to 30 month of age. Growth performance, behavior and carcass quality traits of each steer were recorded and compared between the treatment groups.

**Results:**

In general, the average daily gain during the fattening period was lower in group T2 than in T1 and the control groups. However, carcass weight and dressing percentage was lower in the control group than in T1 or T2 groups (p<0.05). Also, marbling score at 30 months of age was the lowest in the control group (p<0.05), while the three heads group (T2) had the greatest *longissimus* muscle area and marbling score (p<0.05). The behavior of walking time was the greatest in T2 group, while self-grooming and fighting occurred with the most frequency in the control group (p<0.05).

**Conclusion:**

Our results show that the steers in more spacious stocking density had better carcass quality and wellbeing related behaviors, indicating that a lower density has a positive effect on raising management and carcass quality. Thus it is a need to install appropriate pens fitted to welfare-oriented management practices from growing to fattening period in Hanwoo cattle.

## INTRODUCTION

Korean beef consumers prefer Hanwoo beef with high quality to imported beef [1], and many studies have been conducted to improve meat productivity and quality of Hanwoo. It is also important to develop strategies to export Hanwoo meat. Although various factors influence the production of Hanwoo meat [2], the rearing space within the barn, in which an individual is raised from birth until 24 hours prior to shipment, vegetable diet space and stocking density including resting space could be important factors influencing meat quality [3].

Several inhibiting factors under overcrowded rearing conditions could be occurred such as the indoor temperature, changes in humidity within the barn, aggravation of air, depreciation of hygienic state due to excrement, or increased disease contraction ratio along with decreased growth rate owing to the rapid disintegration of nutrients within body, because of social stresses caused by small space between animals [4]. However, the negative factors can be avoided by conducting environmentally friendly rearing, which would then improve meat quality [5,6].

Recently, it is reported that barn facilities have adopted natural air circulation by elevating roof height, or installed automatic feeders, or hen and cow brushes to better manage livestock [7,8]. However, studies on behavior relevant to rearing space have not focused on proper animal stocking density in Hanwoo [9].

Therefore, this study aimed to investigate the effects of different stocking densities on meat production and behavior characteristics in Hanwoo, so as to provide a basis on establishment of management practices to meet the condition of animal welfare and good meat quality.

## MATERIALS AND METHODS

### Animals and experiment design

**Figure 1:**
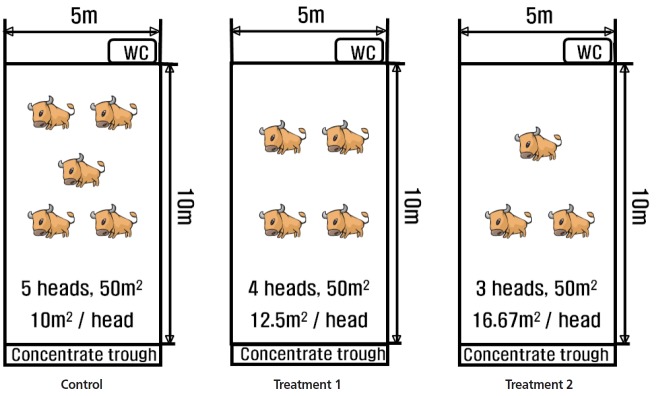
Experimental design of different stoking densities.

Thirty-six Hanwoo steers were randomly chosen in Gyeongbuk Livestock Research Institute from May, 2014 to November, 2015. Preliminary experiments were carried out for three weeks, such that the individuals were grouped by considering average weight. The average yearling weight of the individuals was 291.4±12.2 kg at 12 month of age. The steers were raised in the same rearing area of 50 m^2^ per pen, whereas the stocking density varied (Figure 1). In the pen for the control group (C), a set of five steers of 12 to 30 months of age was assigned, so as to allow 10 m^2^ per head of rearing space. For treatment 1 (T1) and treatment 2 (T2) groups, four and three steers were assigned in a pen, so as to give 12.6 m^2^ and 16.7 m^2^/head rearing space, respectively. Each steer was randomly assigned to each treatment, and three replicates for each treatment were applied, totaling 36 samples (3×5 [C]+3×4 [T1]+3×3 [T2]).

### Animal management

The nutrient composition of feedstuffs is presented in Table 1 [10]. All animals were given the same diets, while forage was restricted to consumption of rice straw. The amount of feedstuffs was changed at three month intervals that were based on the mean body weight.

The fattening stage was divided into two phases: phase I (12 to 21 months of age) and phase II (22 to 30 months of age), and the average amount of concentrate feeding for a day was 5.5 to 10.5 kg per steer, which was approximately equal to 1.6% to 1.8% of body weight (Table 2). During the fattening period, rice straw diets were provided *ad libitum*, twice a day at 8 am and 5 pm, respectively. One water dish and mineral block trough was provided per group, allowing animals to be freely accessed to feed and water.

### Data collection

The total amount of feed intake per steer was recorded by measuring the differences of feed weight provided each day and residual quantity at the end of the next morning (Table 3). Body weight of each steer was measured between 12 and 30 months of age in a three month interval, i.e. before morning feeding at the beginning of each quarter (Table 4). Average daily gain (ADG) between three month intervals was also calculated (Table 5). To evaluate carcass quality traits, a 2 MHz real time ultrasound equipment was applied between the 13th rib and lumber vertebra (HS-2000; HONDA, Tokyo, Japan). The images reflecting ultrasound back fat thickness, *longissimus* muscle area, and marbling score were processed and analyzed using the Image-pro Express (Version 4.1, Media Cybernetics, Rockville, MD, USA) software. Also, all of the steers were slaughtered at a local municipal slaughterhouse at 30 months of age, and carcass quality traits were evaluated according to the Korean carcass grading standard [11].

**Figure 2:**
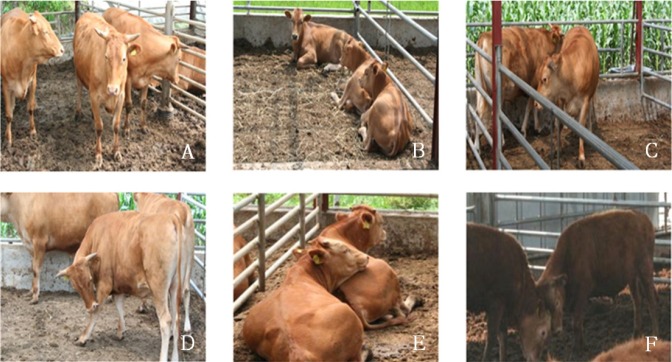
Classification of behavior characteristics. (A) Standing (ST), (B) Lying down (LD), (C) Walking (WA), (D) Self grooming (SG), (E) Leaning (LN), (F) Fighting (FT).

Digital cameras (IR LED camera; Sony, Changwon, Korea) and video-recording devices (STLV-36D; Samsung, Korea) were utilized for precise monitoring of animal behaviors within each treatment group. Nine digital cameras were allocated in front of the feeding area of each pen at a height of approximately 3 m. Animal behavior for a given quarter interval was analyzed by selecting a month and by recording on every weekend of the month. Behavior characteristics were classified as six categories, i.e. standing, lying down, walking, self-grooming, leaning, and fighting (Figure 2), and each individual was observed for 12 hours a day from 7 am to 7 pm. Behavioral time was analyzed by sampling at a two minute interval of the observation records, after which the best behavioral category was determined for each individual [12].

### Statistical analysis

The mean value of the analyzed traits was calculated for each treatment group. Statistical analysis was conducted using the general linear model Procedure, Statistical Analysis System [13] by fitting a linear model with one main effect of different stocking density. Duncan multiple range tests were used to determine statistical significance between treatment means at 0.05 level.

## RESULTS AND DISCUSSION

### Growth performance

Table 3 shows the average of total feed intake per steer. The amounts of feed intake were not significantly different between different space density groups (p>0.05). However, the steers in more spacious groups had marginally more feed intakes, which may be due to more appetite with increasing activity.

Generally T2 group had heavier body weights than the control and T1 groups during the early fattening period (p<0.05) (Table 4). This result supports the report of Boe and Faerevik [14], in which the welfare of livestock was closely related to productivity. Also, Rind and Phillips [15] reported that it was important to provide sufficient space to ensure welfare and productivity of cattle. However, there was no statistical significance of difference of body weight at 30 months of age between the three treatment groups in this study (Table 4). At 15 to 18 months of age, the control group had greater ADG than T1 group (p<0.05), but T1 group had higher ADG than the control and T2 groups at 18 to 21 months of age. This result corresponds to the report of Li et al [16], in which the animals reared with a large group size had significantly greater ADG in an early fattening period. This result reflects strong competition on eating in a large group. As fattening period is extended, the relative space becomes more limited for the steers in denser pens and thus activity of the steers would decrease correspondingly.

In the late fattening period (21 to 30 month), the steers in T2 group, in general, had lowest ADG, followed by T1 and the control group, even if there was no statistical significance between the treatment groups (Table 5).

Li et al [16] reported that the animals reared in a spacious group had greater ADG in an early fattening period. However, our results in this study were not consistent with Li et al [16]. Further, the boy weight at 30 months of age was not significantly heavier in the T2 group (Table 4). This result might be partly due to the fact that the density space (10 m^2^ per steer) in the control group was larger than the average space (7 to 8 m^2^ per steer) in the Hanwoo farms, so that Hanwoo steers have been well adapted to low stocking density pens for the several decades. Also, the average live weight of steers in Hanwoo farms before slaughter was around 700 kg, while our results showed around 680 kg in the control group, i.e. about 3% lower, which might be due to more motility of the steers in spacious stocking density than in Hanwoo farms.

Berg and Butterfield [17] reported that ADG was also affected by differences between individuals, nutritional level of feeds or other various environmental factors. Therefore, further investigation on the relationship between ADG and motility is needed.

### Carcass characteristics

Table 6 shows ultrasound carcass quality traits at different growth stages and at slaughter. In general, T2 group had lower back fat thickness across all growth stages, except at slaughter, even though there was no statistical significance (p>0.05). This may be partly due to greater motility environments in the T2 group compared to T1 and the control groups. Kang et al [18] reported a back fat thickness of 9.9 mm when the Hanwoo steers were slaughtered after rearing for 540 days under the similar management as in this study. They found that all treatment groups showed the greatest fat deposition after 27 months of age. Lee et al [19] reported that there was an increase in back fat thickness in response to an increased level of concentrated feeds during early and late fattening periods, and that back fat thickness increased with unlimited feeding concentrate at 24 months of age.

For the *longissimus* muscle area, T2 group was, in general, the greatest throughout the entire growth period and also at slaughter, i.e. 94.6 cm^2^, followed by T1 group (92.8 cm^2^), and the control group was lowest (91.0 cm^2^), even if statistical significance was limited between the groups, partly due to the small sample size (Table 6). This result was comparable to the report of KOSIS [20], in which the average *longissimus* muscle area of steers that were raised in Hanwoo farms and slaughtered was about 94.3 cm^2^, and are consistent with the report that an increase in motility leads to an increase in musculoskeletal system, thereby improving muscle quantity [21]. The *longissimus* muscle area in all treatment groups increased greatly from 18 to 21 months of age, when the ADG was the greatest. This result corresponds to Kim et al [22], who reported that increases in body weight led to increased *longissimus* muscle area.

The *longissimus* muscle area after slaughter ranged 91.0 to 94.6 cm^2^, which was greater than the result (87.7 cm^2^) of Kang et al [18], for which Hanwoo steers were fed with concentrated feed *ad libitum* during late fattening period. The difference of results between Kang et al [18] and this study may be partly due to different genetics of individuals and rearing factors.

The marbling was nearly absent (score, 1) for all steers before 18 months of age, after which marbling deposition occurred in all treatment groups (Table 6). After 18 months of age, T2 had the greatest marbling score across growth stages, and also at slaughter, while the control group had the smallest score (p<0.05). This result indicates that there is a relationship between fat deposition and motility of steers, i.e. the more spacious in a pen would cause greater motility of steers, such that more deposition of fat between muscles would occur. From the report of Hiroyuki et al [23], it can be inferred that beef cattle consumes large amounts of concentrated feed to convert surplus energy to fat accumulation. During the process of digestion and absorption metabolism for the excess energy of fat, a great deal of oxidative stress occurs in the cell, leading to an increase of active oxygen and nitric oxide and to inhibition of fat cell adiposity.

Table 7 shows the carcass weight and dressing percentage (ratio [%] of carcass weight to final live weight before slaughter) for each stocking density. T1 (423 kg) and T2 (417 kg) groups had heavier carcass weights than the control groups (398 kg) (p<0.05). Also, the dressing percentages for the T1 (62%) and T2 (61%) steers were significantly greater than the control group (58%) (p<0.05). This result supports the report that increasing motility during the fattening periods increased muscle amount, causing greater carcass rate [21].

### Behavior characteristics

Table 8 shows the average time and frequency of the six behavior characteristics in different stocking densities. The time of standing behavior was the longest in the control group, but the shortest for T2 group, while for a resting behavior measure, lying-down, T2 group had the longest time, even if statistical significance was limited (p>0.05). Houpt et al [24] reported that bulls spent 12 hours a day lying down, which means that steers spend much time in a lying-down position. Also, our results showed that the lying-down behavior was opposed to standing behavior. T2 had greater amount of walking behavior than the other two treatment groups (p<0.05). This result makes sense in that more space in the pen enabled the steers to allow more chance of motility. Instead, less time of walking would be caused by smaller space of the pen [25].

Self-grooming behavior may be caused by sanitary purposes or adaptation into the environment [26]. T2 group had the lowest frequency of self-grooming behavior, while the control group had the greatest frequency (p<0.05). Ishi [27] reported that narrow rearing spaces resulted in increased excretion storage areas, causing increased contamination of the fleece and skin, resulting in increased frequency of self-grooming behaviors.

The frequency of fighting was the greatest in the control group, while the lowest in T2 group (p<0.05). According to Kondo et al [28], high stocking density increased fighting behavior patterns such as intimidation, assault or fighting between group members. Our results also revealed more frequent fighting behavior in the control group (Table 8), which was supported by Zeeb et al [29], who reported reduced frequency of fighting behavior as stocking density decreased. Jensen et al [30] also reported that fighting behavior was affected by stocking density.

## IMPLICATIONS

This study described the relationships between stocking density and growth and carcass quality traits as well as behavior characteristics in Hanwoo, and indicated positive effect of the spacious stocking density on carcass quality and behavior characteristics. Even if the samples to be used in this study were not large enough to give more definite evidence, our results point out the importance of spacious rearing conditions in improving meat quality traits of Hanwoo. Further study is needed to verify implementation of favorable pens to Hanwoo cattle to consider a positive effect on raising management and carcass quality, so as to fit to welfare-oriented management practices from growing to fattening period.

## Figures and Tables

**Table 1:** Composition of nutrients in the experimental feedstuffs for Hanwoo steers

Nutrients (%)	Concentrate		Rice straw

	Early fattening	Late fattening	
Dry matter	83.55	83.18	90.09
Crude protein	14.19	12.31	4.53
Crude fat	3.49	4.87	2.17
Crude fiber	4.57	4.25	27.66
Crude ash	4.85	5.90	14.31
Calcium	0.72	0.94	0.22
Phosphorous	0.42	0.35	0.12
Total digestible nutrients	72.43	73.75	37.13

**Table 2:** The amounts of feed intake in the two main fattening phases

Month		Feed intake (kg/head/d)	

		Concentrate	Rice straw
Phase I (Early fattening)	12	5.5	3.5
	13	6	3.5
	14	6	3.5
	15	6.5	3
	16	7	3
	17	7	3
	18	7.5	2.5
	19	8	2.5
	20	8	2.5
	21	8.5	2
Phase II (Late fattening)	22	8.5	2
	23	9	2
	24	9	1.5
	25	9.5	1.5
	26	9.5	1.5
	27	10	1
	28	10	1
	29	10.5	1
	30	10	0.5

**Table 3:** The average amounts of feed intake (kg/head) in different stocking densities in Hanwoo steers

Feeds	Stocking density group		

	Control1)	Treatment 12)	Treatment 23)
Total	5,386±47	5,403±47	5,430±47
Concentrate	4,193±21	4,198±21	4,212±21
Rice straw	1,193±14	1,205±14	1,218±14

1) 10 m^2^/head.

2) 12.5 m^2^/head.

3) 16.7 m^2^/head.

**Table 4:** Effects of different stocking density on body weight (kg) in Hanwoo steers

Month	Treatments		

	Control1)	Treatment 12)	Treatment 23)
12	283.8±16.7	292.3±16.0	298.3±16.2
15	351.6±13.8^b^	362.8±11.5^ab^	370.7±10.7^a^
18	417.3±11.6^ab^	412.1±12.3^b^	438.4±10.6^a^
21	508.2±13.1^b^	519.5±11.2^ab^	525.3±12.4^a^
24	560.0±20.3	575.0±16.8	581.3±17.1
27	631.0±18.6	639.7±14.0	644.3±18.4
30	683.5±21.3	680.6±27.0	683.7±25.5

1) 10 m^2^/head.

2) 12.5 m^2^/head.

3) 16.7 m^2^/head.

a,b Means with different superscripts in the same row differ significantly (p<0.05).

**Table 5:** Effects of different stocking density on average daily gain (kg/d) in Hanwoo steers

Month	Treatments		

	Control1)	Treatment 12)	Treatment 23)
12–15	0.75±0.16	0.78±0.03	0.76±0.14
15–18	0.73±0.09^a^	0.55±0.04^b^	0.64±0.07^ab^
18–21	1.01±0.19^ab^	1.19±0.06^a^	0.96±0.09^b^
12–21	0.83±0.08	0.84±0.09	0.84±0.12
21–24	0.58±0.13	0.62±0.04	0.62±0.03
24–27	0.79±0.14	0.72±0.10	0.70±0.01
27–30	0.58±0.08	0.46±0.21	0.44±0.19
21–30	0.65±0.14	0.60±0.12	0.59±0.11
12–30	0.74±0.08	0.72±0.07	0.71±0.05

1) 10 m^2^/head.

2) 12.5 m^2^/head.

3) 16.7 m^2^/head.

a,b Means with different superscripts in the same row differ significantly (p<0.05).

**Table 6:** Effects of different stocking density on ultrasound and carcass quality traits of Hanwoo steers

Items	Months	Treatments		

		Control1)	Treatment 12)	Treatment 23)
BFTU (mm)	12	1.50±0.93	1.13±0.34	1.21±0.59
	15	1.75±1.16	1.25±0.45	1.29±0.75
	18	2.63±1.69	1.94±0.93	2.33±1.27
	21	4.00±2.33	3.56±1.15	3.92±1.38
	24	4.75±2.60	4.56±1.63	4.71±1.82
	27	6.25±3.01	6.25±2.27	6.13±2.02
BFTC (mm)	30	9.63±3.02	9.75±2.72	10.26±2.47
LMAU (cm^2^)	12	42.86±3.61	46.03±4.20	45.79±3.86
	15	48.11±3.77	49.92±3.59	50.93±4.17
	18	60.16±3.58	60.63±3.35	62.56±3.40
	21	72.59±3.83	71.90±3.54	72.86±4.60
	24	77.56±2.68	77.83±3.31	78.09±4.39
	27	84.00±5.38	85.72±4.69	85.93±4.35
LMAC (cm^2^)	30	91.00±6.68	92.81±6.80	94.55±6.66
MSU	12	1.00±0.00	1.00±0.00	1.00±0.00
	15	1.00±0.00	1.00±0.00	1.00±0.00
	18	1.88±0.99	2.06±0.85	2.67±1.13
	21	2.63±1.06	2.25±1.06	2.96±1.40
	24	3.75±1.16	4.00±1.26	4.42±1.44
	27	5.00±1.98	5.38±1.82	6.13±1.58
MSC	30	5.25±1.57^b^	6.13±1.35^a^	6.63±1.84^a^

BFTU, ultrasound back fat thickness; BFTC, carcass back fat thickness; LMAU, ultrasound *longissimus* muscle area; LMAC, carcass *longissimus* muscle area; MSU, ultrasound marbling score; MSC, carcass marbling score.

1) 10 m^2^/head.

2) 12.5 m^2^/head.

3) 16.7 m^2^/head.

a,b Means with different superscripts in the same row differ significantly (p<0.05).

**Table 7:** Effects of different stocking density on carcass weight and dressing percentage in Hanwoo steers

Items	Treatments		

	Control1)	Treatment 12)	Treatment 23)
Carcass weight (kg/head)	397.8±11.7^b^	423.4±16.9^a^	417.0±15.3^a^
Dressing percentage (%/head)	58.2±1.7^b^	62.2±2.6^a^	61.0±2.2^a^

1) 10 m^2^/head.

2) 12.5 m^2^/head.

3) 16.7 m^2^/head.

a,b Means with different superscripts in the same row differ significantly (p<0.05).

**Table 8:** Effects of different stocking density on behavior characteristics in Hanwoo steers

Items	Control1)	Treatment 12)	Treatment 23)
Minute			
Standing	221.6±25.8	209.5±19.4	207.1±27.9
Lying down	119.8±27.8	128.3±34.1	130.6±20.3
Walking	14.5±7.7^b^	11.6±3.5^b^	34.8±17.4^a^
Count			
Self grooming	24.28±20.44^a^	10.30±8.12^ab^	7.36±11.08^b^
Leaning	5.41±1.86	4.91±0.76	4.27±1.13
Fighting	6.56±2.12^a^	4.89±2.71^ab^	3.24±2.66^b^

1) 10 m^2^/head.

2) 12.5 m^2^/head.

3) 16.7 m^2^/head.

a,b Means with different superscripts in the same row differ significantly (p<0.05).
